# Bioengineering Strategies for Protein-Based Nanoparticles

**DOI:** 10.3390/genes9070370

**Published:** 2018-07-23

**Authors:** Dennis Diaz, Andrew Care, Anwar Sunna

**Affiliations:** 1 Department of Molecular Sciences, Macquarie University, Sydney, NSW 2109, Australia; dennis.diaz-rincon@hdr.mq.edu.au (D.D.); andrew.care@mq.edu.au (A.C.); 2 Australian Research Council Centre of Excellence for Nanoscale BioPhotonics, Macquarie University, Sydney, NSW 2109, Australia; 3 Biomolecular Discovery and Design Research Centre, Macquarie University, Sydney, NSW 2109, Australia

**Keywords:** protein-based nanoparticles, bioengineering, nanobiotechnology, synthetic biology, biomedicine, biocatalysis, virus-like particle, nanocages

## Abstract

In recent years, the practical application of protein-based nanoparticles (PNPs) has expanded rapidly into areas like drug delivery, vaccine development, and biocatalysis. PNPs possess unique features that make them attractive as potential platforms for a variety of nanobiotechnological applications. They self-assemble from multiple protein subunits into hollow monodisperse structures; they are highly stable, biocompatible, and biodegradable; and their external components and encapsulation properties can be readily manipulated by chemical or genetic strategies. Moreover, their complex and perfect symmetry have motivated researchers to mimic their properties in order to create de novo protein assemblies. This review focuses on recent advances in the bioengineering and bioconjugation of PNPs and the implementation of synthetic biology concepts to exploit and enhance PNP’s intrinsic properties and to impart them with novel functionalities.

## 1. Introduction

Protein-based nanoparticles (PNPs) are present in all three domains of life, where they form highly organized supramolecular structures with unique biophysical properties. PNPs are composed of multiple copies of one or more types of monomeric protein building blocks (subunits), which self-assemble into highly organized hollow structures that are 10–100 nm in diameter [1]. Many PNPs exhibit sphere-shaped conformations with polyhedral symmetries, although some other shapes have been reported [2,3]. They possess at least one internal cavity and static and/or gated pores located in between protein subunits that give access to the cavity [4]. In nature, the internal cavities provide confined spaces that act as containers for enzymes and their substrates and byproducts; storage compartments for minerals; chaperones for the sequestration of partially unfolded proteins; and protective carriers for genetic material (e.g., viruses) [5,6].

Protein-based nanoparticles have many other useful attributes that make them highly attractive as biological nanomaterials. For instance, (i) they are soluble, monodisperse, biocompatible, and have robust structures [7]; (ii) they have three different interfaces that can be engineered to gain new functionalities (internal, external, and inter-subunit) [8]; (iii) the crystal structure, genetic, and molecular information of many PNPs are available, allowing for rational chemical and/or genetic modifications to be performed [9,10]; (iv) their uniform and highly repetitive structures enable the homogeneous incorporation and display of multiple copies of a moiety(ies) [11]. Additionally, scientists have gained new insights into the unique structural characteristics and self-assembly mechanisms of natural PNPs, which have informed the design and construction of novel synthetic PNPs from natural oligomeric proteins and/or in silico designed protein sequences [12].

The ability of PNPs to encapsulate a diverse range of molecular cargoes (e.g., catalytic, therapeutic, or imaging agents) and their amenability to functionalization have to led to their use in numerous practical applications [13]. Herein, we review the new and existing synthetic biology tools used to design and engineer functional PNPs, and also highlight their application in vaccine development, drug delivery, and biocatalysts.

## 2. Identification, Production, and Purification of Protein-Based Nanoparticles

The identification of PNPs has become easier due to the accessibility of genomic data and the development of powerful bioinformatics software for genome mining. For example, Giessen and Silver used this combined approach to identify 900 putative encapsulin systems in bacterial and archaeal genomes [14]. However, it should be noted that only a limited number of PNPs have been studied in-depth, including nonviral PNPs like ferritin, heat shock proteins (Hsp), DNA-binding proteins from starved cells (Dps), encapsulin, the E2 protein of pyruvate dehydrogenase, lumazine synthase, vault proteins, and virus-like particles (VLPs, which resemble viruses, but contain no viral genetic material and are therefore non-infectious) [15]. The structural features and natural functions of the most studied PNPs are summarized in Table 1.

Protein-based nanoparticles have been produced either in their natural hosts, as recombinant proteins in expression systems (e.g., bacteria, yeast, plants, and insect or mammalian cells), or by cell-free protein synthesis. As shown in Table 1, the majority of PNPs described in this review have been expressed and produced recombinantly in *Escherichia coli*. However, the functional expression of eukaryotic PNPs in *E. coli* remains a challenge because they often require complex protein folding and post-translational modifications [16]. For instance, due to these constraints, vault proteins and the cowpea mosaic virus (CPMV) VLP cannot be produced in *E. coli* and instead require insect cells and/or plant expression systems [17,18]. Selecting the most suitable production method can make a significant difference when it comes to achieving high production yields for PNPs. For example, the norovirus VLP exhibits low production yields in *E. coli* (1.5–3 mg/L). Expression of the same VLP in the yeast *Pichia pastoris* improved its yield by 200-fold, but production times increased (~50 h). Similar yields were then obtained within only four hours using a cell-free protein synthesis system (based on *E. coli* lysate) [19].

Due to their large macromolecular structures, PNP purification protocols tend to involve size exclusion chromatography (SEC) and/or differential centrifugation (e.g., sucrose gradient) steps [20,21]. To achieve higher PNP purity, SEC is generally combined with affinity chromatography, in which the PNP displays a pre-selected purification tag (e.g., histidine-tag), or ion-exchange, in which the outer surface charge of the PNP is exploited [22,23]. PNP purification protocols often require protein concentrating steps with polyethylene glycol (PEG, e.g., PEG8000) or ammonium sulfate precipitation [24,25]. In addition, depending on the thermal stability of a PNP, an initial heat treatment step (>60 °C) can be performed to precipitate the majority of production host proteins prior to any chromatographic techniques [26,27].

## 3. Rational Design of Protein-Based Nanoparticles 

In the redesign of natural protein assemblies, it is important to understand how proteins fold and maintain their structure, and how their natural arrangement relates to their function. Protein-based nanoparticles exhibit complex, yet genetically modifiable protein architectures. The genetic, molecular, and structural (including crystal structures) data of some PNPs are readily accessible. This information, in conjunction with new computational tools for molecular design and engineering, makes PNPs ideal candidates for rational redesign [67]. Ferritins are one of the most well-studied PNPs and are regularly engineered to have enhanced or new functionalities. Kim and co-workers used 3D modeling and simulation tools (e.g., Modeller V 9.19, PEP-FOLD, and Pymol) to predict the length of an “intrinsically disordered peptide” (referred to as XTEN) required to optimally cover the surface of ferritin [68]. Ferritin subunits displaying C-terminal XTENs (with differing lengths) and their capacity to assemble into ferritin PNPs was simulated in silico. The four best XTEN-displaying ferritin variants were selected and successfully expressed in *E. coli*. The variants were further modified to display targeting peptides and affibodies for pharmacokinetic studies in vivo (mice). The engineered ferritin variants showed superior binding avidity and selectivity, enhanced pharmacokinetic profiles, slower clearance rates, and improved targeting, which are important aspects in therapeutic applications (e.g., drug delivery). This study clearly demonstrates the potential of rational design tools to optimize the modification of PNPs, thus enhancing their functionality in a specific application [68,69].

Redesigning native protein assemblies can be challenging because they have evolved to be marginally stable, meaning that even the subtlest modifications to their protein sequence can lead to protein unfolding and/or aggregation [70,71,72]. Accordingly, studies using rational design to alter PNPs’ natural properties, such as stability under different conditions (e.g., pH, temperature, and ionic strength), pore size, shell size, and shape, are less common. The rational design of artificial hollow PNPs can be generally achieved via three methods: the fusion approach; domain swapping; and metal-directed self-assembly. In the fusion approach, two (or more) different proteins that are individually capable of self-assembling into oligomers are fused together via a small linker domain that matches the chosen symmetry. Thus, identical copies of the resulting fusion protein self-assemble into the pre-designed particle or biomaterial [73,74]. This strategy was used by Lai et al. to construct tetrahedral PNPs from two different oligomeric protein domains (a dimer and a trimer) joined together by an α-helical linker [75]. In this study, an idealized molecular model was created in silico to help identify potential mutation sites to improve the PNP’s structural asymmetry. In the domain-swapping approach, a secondary structural domain of one protein is substituted with the corresponding domain located within another protein, resulting in an intertwined oligomer [76]. Metal-directed self-assembly involves the construction of new protein interfaces that arise from the coordination of metal ions that have large binding energies [77]. Employing these two strategies, Miyamoto et al. obtained a novel C-type cytochrome (Cyt *cb*_562_) cage structure, in which three domain-swapped Cyt *cb*_562_ dimers formed a cage by the coordination of amino acids to Zn^2+^ metal ions [76].

Coiled-coil peptides are structural motifs within proteins that bind one another via hydrophobic interactions, thus mediating protein oligomerization. Consequently, coiled-coil peptides have been used in the development of new PNPs. For example, Fletcher et al. described de novo coiled-coil peptides designed to form two different oligomer states that, once mixed, were able to arrange into ~100 nm unilamellar self-assembling cage-like particles (SAGEs) [78].

**Figure 1:**
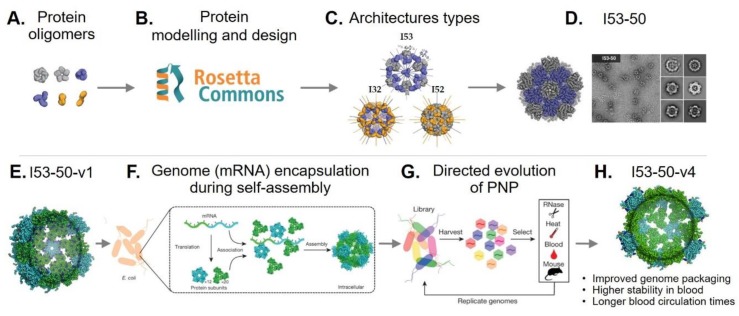
Rational design and directed evolution of a synthetic nucleocapsid. (**Upper panel**) Diagram showing the in silico design process and subsequent production of a de novo PNP. (**A**) Pentameric (gray), trimeric (blue), and dimeric (yellow) protein oligomers selected for rational design. (**B**) The Rosetta software was employed in the in silico design of new PNPs. (**C**) Rosetta-predicted PNP architecture types. (**D**) Design model I53-50 and its corresponding negative-stain electron micrograph, showing that the obtained structure matched the predicted in silico model. Adapted with permission of [80]. (**Lower panel**) Synthetic nucleocapsid design and evolution. (**E**) Model of the I53-50-v1. Pentamers (cyan) and trimers (green). (**F**) Nucleocapsids are capable of encapsulating their own genome (mRNA) during self-assembly in *Escherichia coli.* (**G**) *E. coli* is transformed with a library of synthetic nucleocapsid variants. All variants are purified together from cell lysates and selected against RNase, heat, blood, and in vivo blood circulation. The mRNA inside the selected capsids variants is then obtained and amplified using quantitative reverse transcription PCR (RT-qPCR), re-cloned to construct a new library, and transformed into *E. coli* for another round of selection. (**H**) After several rounds of evolution, an improved version of the original nucleocapsid was obtained (I53-50-v4). Adapted with permission of [81].

Recent advances in computational design have allowed the de novo design of protein–protein interfaces [73]. The Baker’s lab has developed the Rosetta software as a computational tool for protein structure prediction and design [79]. Using Rosetta, they redesigned dimeric, trimeric, and pentameric protein oligomers (Figure 1A,B) to act as building blocks for the assembly of megadalton-scale icosahedral PNPs. To achieve this, three architectural types (I53, I52, and I32) were formed from pairwise combinations of the dimeric/trimeric and pentameric oligomers (Figure 1C). These were then used to model novel PNPs by fitting protein oligomers with similar crystal structures in the Protein Data Bank (PDB, http://www.rcsb.org/pdb/) or de novo designed protein oligomers. The resulting designs were sorted based on different selection criteria, including interface area, predicted binding energies, and shape complementarity. A number of designs were selected and expressed recombinantly in *E. coli*, and those capable of self-assembly into PNPs were further characterized. Ten designs were found to self-assemble and form PNP structures (26–31 nm diameters) that matched their predicted/modeled architectures. The de novo designed I53-50 (Figure 1D) exhibited superior single-subunit stability and assembly kinetics similar to those of viral capsids [80].

In more recent work, additional positively charged residues were incorporated into the internal cavity surface of I53-50, allowing it to package its own negatively charged mRNA genome (Figure 1F), thus generating a synthetic “nucleocapsid” (I53-50-v1) (Figure 1E). To determine whether the nucleocapsid could be evolved to acquire more virus-like properties, combinatorial libraries of nucleocapsid variants were produced in *E. coli*. The variants were subjected to sequential rounds of selection (Figure 1G), first against RNase for enhanced genome packaging (more than 133-fold higher); second to blood and RNase at body temperature (37 °C) for increased stability in blood (from less than 3.7% to 71% of the packaged RNA was protected from degradation following six hours of treatment) and in vivo (mice) to increase blood circulation times (from less than 5 min to over 4.5 h) (Figure 1H). This study shows that PNPs can be de novo designed and that their properties can be evolved further, thus providing an exciting opportunity to create non-natural PNPs with novel functionalities [81].

## 4. Bioengineering Functional Protein-Based Nanoparticles 

**Figure 2:**
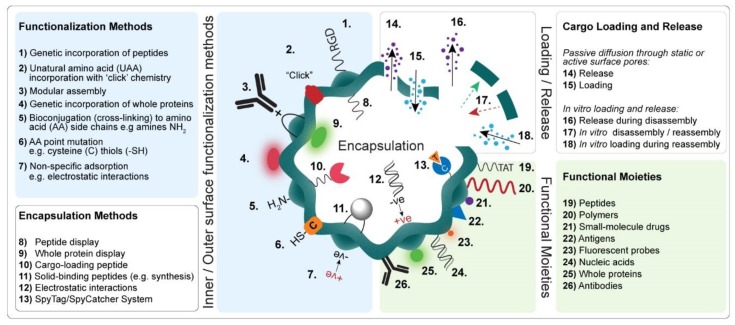
Illustration showing the various approaches used to functionalize PNPs with different functional moieties, and the strategies employed to encapsulate, load, and release cargo molecules. RGD, cell-binding motif (Arg-Gly-Asp); TAT, human immunodeficiency virus (HIV-1) cell-penetrating peptide (CPP); +ve, positive; –ve: negative.

The functional properties of a PNP can be custom engineered to meet the requirement of a particular application by modification of their interfaces via bioconjugation and/or genetic engineering (see Figure 2). Since the genetic, structural, and biochemical properties of many PNPs are well known, this information can be used to design and select the best functionalization strategy for a specific PNP and its intended application [9,10]. In this section, we provide an overview of various bioconjugation and genetic engineering strategies (and combinations thereof) used to functionalize PNPs for a diverse range of bioapplications. Table 2 summarizes the functionalization methods, encapsulation strategies, and reported cargo molecules for a selection of PNPs.

### 4.1. Bioconjugation

The functionalization of PNPs using bioconjugation strategies is effective for the attachment of various moieties that cannot be introduced via genetic modification. This includes large moieties (e.g., antibodies) that may disrupt PNP self-assembly, and non-biological moieties (e.g., small-molecule drugs) that cannot be produced in heterologous hosts (e.g., *E. coli*) [7,82].

#### 4.1.1. Noncovalent Bioconjugation of Protein-Based Nanoparticles 

The nonspecific physical adsorption of moieties onto PNP surfaces requires no complex chemical reactions and has been used to encapsulate cargoes and/or functionalize PNPs outer surfaces. For example, cationic polymers like polyamidoamine (PANAM) have been electrostatically bound to the negatively charged cowpea chlorotic mottle virus (CCMV) VLP capsid, resulting in the formation of hexagonal higher-order structures. Large-sized polymers (>20 positive charges) maintained their affinity towards the VLP at high salt concentrations. However, under the same conditions, midsized polymers (8–9 positive charges) lost their affinity, while small-sized polymers (<4 positive charges) could not form complexes even at low salt concentrations [83]. Although the simplicity of physical adsorption is an attractive strategy to functionalize PNPs, it relies on weak noncovalent interactions that are easily disrupted and may cause the disassociation of moieties from the PNP surface in complex fluids (e.g., blood).

#### 4.1.2. Covalent Bioconjugation of Protein-Based Nanoparticles 

The covalent bioconjugation of functional moieties to PNPs is achieved by exploiting reactive groups of the naturally occurring amino acids exposed on their inner or outer surfaces. This approach forms highly stable bonds leading to strong irreversible interactions between the involved molecules [84]. Commonly used crosslinking agents in the bioconjugation of PNPs include maleimides for cysteine thiols; *N*-hydroxy-succinimides (NHS) for lysine amines; and carbodiimides for glutamate or aspartate carboxylates [85]. These agents have been extensively applied to bioconjugate PNPs to antibodies [86], fluorescent dyes [87,88], folic acid [89], cell-targeting and cell-penetrating peptides [90], and small-molecule drugs [91]. However, bioconjugation techniques often suffer from poor selectivity and low reproducibility and can result in moieties attached with altered conformations and random orientations, causing a reduction/loss of their function [13,92]. Furthermore, some bioconjugation processes are highly complex and require abrasive chemical reagents (e.g., organic solvents) and high temperatures, which may reduce a PNP’s stability.

Recently, click chemistry reactions have been shown as a viable functionalization technique for PNPs. This strategy denotes a set of reactions that are fast, straightforward, specific, and efficient. The copper-catalyzed azide–alkyne cycloaddition (CuAAC) reaction is a reliable technique that relies on the presence of alkynes and azides in the PNP and the moiety to conjugate [93]. These reactive groups have been introduced into PNPs mainly by residue-specific replacement using amino acid analogues (e.g., azidohomoalanine or homopropargylglycine) and unnatural amino acids (UAAs) [94]. This approach has been used by Finn and co-workers to bind carbohydrate-based ligands, fluorogenic dyes, proteins, and polymers to Qβ VLP [95,96,97].

### 4.2. Genetically Engineered Protein-Based Nanoparticles 

Genetic modification of PNPs allows for the precise control over the number, position, and distribution of incorporated moiety(ies) [11]. Accordingly, genetic modification alone or in combination with bioconjugation techniques (as described above) is fast becoming the preferred strategy for engineering functional PNPs.

#### 4.2.1. Peptide and Protein Display

Functional peptides or proteins can be genetically fused to the N- or C-terminus, or within loop regions of PNP subunits that are known to be exposed on their outer surface. Some examples include: the N-terminus of the lumazine synthase from *Brucella* spp., which has been fused to immunogenic peptides [98]; the C-terminus of the Hsp from *Methanococcus jannaschii* fused to the tumor-targeting peptide RGD-4C (Cys-Asp-Cys-Arg-Gly-Asp-Cys-Phe-Cys) [87]; and the loop at the 42 position of encapsulin, which was fused to a His-tag [99].

Recently, the functional display of whole proteins on the outer surface of PNPs has been reported [100,101]. For example, atomic structural analysis of a grapevine fanleaf VLP revealed several amino acid residues at the C-terminus of the VLP’s subunit that were externally exposed and not involved in any of the protein–protein interactions integral to the VLP’s self-assembly. This region was then modified to successfully display red or green fluorescent proteins on the outer surface of the VLP without disrupting its assembly and stability [100]. Similarly, the C-terminus of de novo designed T33-21 subunit was genetically fused to two different antifreeze proteins, resulting in their individual functional display on the assembled PNP’s outer surface. Both antifreeze PNPs exhibited improved antifreeze activities relative to their monomeric counterparts [101].

#### 4.2.2. Modular Assembly

The genetic incorporation of large moieties into PNPs can disrupt their self-assembly and stability and is therefore limited to peptides and small proteins. To overcome this, modular assembly strategies have been developed that allow the indirect attachment of large moieties to the outer surfaces of PNP surfaces. For example, a peptide that binds the fragment crystallizable (Fc)-region of antibodies has been introduced onto the outer surface of ferritin using genetic engineering. This Fc-binding peptide was then shown to act as an effective anchorage point for the modular attachment of large and structurally complex immunoglobulin G (IgG) antibodies to the PNP [102].

Another modular assembly strategy for antibody display is based on the high affinity of the streptavidin (StAv)–biotin binding system, which is widely used in biology and medicine as a molecular adaptor. Currently, biotin can be covalently linked to a variety of molecules (e.g., proteins, peptides, nucleotides, carbohydrates, metals), and these biotinylated molecules bind to StAv with high affinity to form a conjugate [103]. This approach was used to develop a functionalized Janus PNP [104]. Briefly, a tetrapeptide was genetically fused to Dps from *Listeria innocua* to be displayed externally. The tetrapeptide was employed to immobilize Dps to thiol-reactive beads. Then, biotin was linked to the accessible tetrapeptides on the Dps surface for subsequent StAv binding. The resulting StAv-functionalized Dps PNPs were released from the beads, leaving the non-biotinylated subunits available for further functionalization. This functionalization platform allows the attachment of any biotinylated moiety to the PNP, enabling various applications. For example, targeting of *Staphylococcus aureus* was achieved by a fluorescein-labeled StAv-functionalized Dps, which displayed a biotinylated monoclonal antibody against protein A expressed on the cell surface of *S. aureus*.

A similar approach to modular incorporation of molecules is enzymatic labeling with sortase A. Sortases are found in Gram-negative bacteria, where they are crucial in the covalent binding of proteins to the peptidoglycan cell wall. Sortase A from *S. aureus* can catalyze the peptide bond formation between a protein containing a Leu-Pro-X-Thr-Gly (LPXTG) motif and a peptide with a polyglycine sequence (at the N-terminus). The latter can be decorated with any molecule accessible to chemical synthesis (e.g., fluorophores, crosslinkers, and biomolecules) or other recombinant proteins or peptides [105,106]. This allows for the site-specific modification of proteins at either the N- or C-terminus or internal loops and the formation of cyclized (poly)peptides [106]. For example, sortase A-mediated bioconjugation has been used to attach thermoresponsive biopolymers, cellulose-degrading enzymes, fluorescent proteins, antigens, and fluorescent probes to a range of PNPs [82,107,108]. This technique represents a viable alternative to purely chemical crosslinking or genetic engineering methods and offers several advantages. These advantages include the ability to perform reactions under mild physiological conditions, fewer modification requirements on the target protein, and, since sortases that recognize different amino acid motives are available, it can be orthogonal. Nevertheless, although there is a minimal requirement for prior genetic modification of the protein of interest, several factors potentially affecting the efficiency of labeling, such as flexibility, accessibility, and intrinsic structure of the target region for modification, should be considered [106].

Genetic engineering of PNPs can be used also to improve the specificity and uniformity of bioconjugation strategies by introducing point mutations, aimed at changing and/or removing standard reactive residues, or to introduce unnatural amino acids (UAAs) for subsequent chemical conjugations. Generally, Cys and/or Lys residues are either introduced or removed from the surface of PNPs to improve the selectivity of covalent bioconjugation and to better control the number of functional moieties to be displayed on their surfaces. For example, the incorporation of Cys allowed the thiol-mediated attachment of chromophores, nanogold, and biotin onto the external surface of CMPV [109]. Additionally, mutating natural Cys to other nonreactive amino acids can be used to eliminate nonspecific functionalization that may hinder the PNP macrostructure. An example of this was the substitution of one intrinsic Cys, located at the interface between encapsulin subunits, that could make the structure unstable upon bioconjugation of the anticancer drug doxorubicin (DOX) to an externally displayed Cys [9].

Another strategy to broaden the reactive groups for functionalization beyond the 20 naturally occurring amino acids is the incorporation of UAAs. Generally, two approaches can be used for the metabolic incorporation of UAAs into proteins, namely the residue-specific and the site-specific strategies. Residue-specific methods involve the global (or partial) replacement of the standard amino acid, not just in the protein of interest but in all proteins within the host system [110]. This approach relies on the structural resemblance between the analogue and the standard amino acid. However, other approaches can be applied to improve the incorporation of the analog UAA, such as overexpression or mutations of aminoacyl-tRNA synthetases and the reassignment of sense codons [111]. The site-specific method allows replacement of a single residue while maintaining access to the other 20 standard amino acids. In this approach, the incorporation of the UAA requires an evolved transfer RNA (tRNA)/aminoacyl-tRNA synthetase pair able to first, respond to a nonsense (e.g., amber codon) or four-base codon, and second, recognize the desired UAA [110,112]. The amber codon suppression is one of the most popular and well-stablished techniques for UAA insertion into proteins. This approach has been used successfully by Francis and collaborators to introduce aminophenylalanine into the MS2 VLP, mediating the covalent bioconjugation of fluorophore-labeled aminophenol-containing DNA, DNA aptamers, and antibodies [20,113,114].

#### 4.2.3. Encapsulation of Foreign Cargoes

The internal cavities of PNPs have the capacity to encapsulate, carry, and protect both native and non-native molecular cargo. Molecular cargo are typically loaded inside PNPs via (i) diffusion, in which cargo molecules move through static (i.e., small molecules like ions, water, organic molecules) or gated pores (which open and close in response to specific conditions) located at the interfaces between PNP subunits that give access to the internal cavity [4]; (ii) in vitro loading, in which a disassembled PNP undergoes reassembly in the presence of the molecular cargo, thus encapsulating it; or (iii) in vivo loading, in which molecular interactions between the internal cavity surfaces of a PNP and cargo components facilitate encapsulation during PNP in vivo self-assembly [115]. When selecting the best strategy to load cargo into a PNP, it is important to consider the size of the cargo in relation to the size of the outer pores and/or internal cavity. Another important aspect is potential leakage of the cargo (e.g., via pores) after encapsulation and the possible nonspecific interactions between the cargo and the PNP’s outer surface, which may prevent efficient loading.

Optimization of the loading process conditions should be considered to favor the accessibility of the cargo to the PNP’s inner cavity (e.g., disassembly/reassembly conditions) and to avoid the cargo’s instability. Finally, the stability of the cargo and PNPs under storage and application conditions should be compatible. In this section, we focus on other genetic modifications that can be used to aid the loading of molecules inside PNP cavities that have not been discussed in previous sections of this review.

In nature, the surface of the interior cavity of PNPs often displays an intrinsic affinity towards their natural cargo molecule, enabling the encapsulation of molecules with similar properties [1]. For instance, some VLPs have positively charged interior surfaces, and their electrostatic interactions with negatively charged nucleic acid enhance the self-assembly of the capsid subunits around its natural cargo (i.e., RNA/DNA). This mechanism has been exploited to encapsulate negatively charged non-native cargo into empty capsids (i.e., VLPs), including nonviral RNA, polyanions, and nanoparticles (NPs) (e.g., thiolalkylated tetraethylene glycol-coated gold NPs) [39]. Furthermore, the charge of a non-native cargo can be specifically modified to facilitate its encapsulation within some PNPs, thus eliminating the need for any PNP modification. For example, the positively charged DOX has been co-encapsulated with polystyrenesulfonic acid (PSA). Polystyrenesulfonic acid is negatively charged and mediates the loading of DOX into the positively charged interior of the hibiscus chlorotic ringspot virus VLP [116].

Other PNPs show more complex in vivo loading mechanisms for the encapsulation of their native cargo which are based on specific interactions between interior surface regions and unique components of their native cargoes. These mechanisms have been adapted to facilitate the loading of non-native molecules. For example, the encapsulation of MS2 genomic RNA can be attained via noncovalent interactions between the capsid subunits and the 19-nucleotide specific MS2 cistron, known as the pac site. In the case of Qβ phage, this process is mediated by a 29-nucleotide RNA hairpin, known as the Qβ hairpin [39,44]. The fusion of the pac site and Qβ hairpin to therapeutically relevant microRNAs (miRNAs) and interference RNAs (iRNAs) has allowed their encapsulation inside MS2 and Qβ, respectively [24,90].

Similarly, certain non-virus derived PNPs present unique encapsulation mechanisms. For instance, enzymes that are naturally targeted inside encapsulin can be encapsulated via a C-terminal extension of 30–40 amino acids, referred to as a cargo-loading peptide (CLP). The CLP selectively binds to hydrophobic pockets located on the cavity surface of encapsulin [21]. Foreign cargo proteins, such as fluorescent proteins and enzymes (e.g., firefly luciferase and Aro10p pyruvate decarboxylase) tagged with full or truncated CLP sequences, have been loaded successfully inside encapsulins in vivo and/or in vitro [49,50,51,188].

The mammalian vault protein comprises three proteins—a major vault protein (MVP), a vault poly(ADP-ribose) polymerase (VPARP), and a telomerase-associated protein (TEP1). The vault macrostructure is formed by MVP, which in solution shows transient open and closed states (“breathing” mechanism), allowing the encapsulation of the other two vault-associated proteins [178]. It has been shown that a C-terminal minimal interaction domain (mINT) of 162 amino acids, which binds to MVP’s inner surface, allows the encapsulation of VPARP [179]. Rome and co-workers have genetically fused the mINT domain to various proteins (i.e., green fluorescent protein (GFP), luciferase, chlamydial epitopes, antitumor cytokines and antigens), allowing their in vitro loading into MVP [179,180,181,182,189]. To further extend the loading properties of mINT to inorganic compounds, a gene construct of mINT was designed to contain an N-terminal 31-amino acid extension that included a His-tag. The His-tag in the protein was used to bind gold-Ni-nitrilotriacetic acid (NTA) particles, and the resulting complex was successfully internalized inside the vault capsule. This mINT–gold complex can potentially capture any His-tagged protein and facilitate their loading inside the vault protein, thus avoiding any need for prior genetic fusion of cargo proteins to mINT [183].

Further studies have involved the encapsulation of hydrophobic drugs inside vaults via the fusion of a small amphipathic α-helix derived from the hepatitis C viral nonstructural protein 5A (NS5A) to the N-terminus of MVP, which is exposed on the inner surface. This modification creates a lipophilic setting in the vault protein’s cavity, which allows the reversible association of several hydrophobic therapeutic molecules, including retinoic acid, amphotericin B, and bryostatin 1. However, no association was observed with the hydrophilic drug DOX [184]. Recently, Shang et al. reported that the fusion of NS5A to the C-terminus of hepatitis B virus subunit (HBc) improved the encapsulation capability of the hydrophobic form of DOX inside HBc [134], confirming the properties of NS5A to bind and facilitate the loading of hydrophobic therapeutic compounds inside PNPs.

In another approach, the Douglas group used the intrinsic features of the P22 VLP to develop a supramolecular platform for several applications ranging from material sciences to biomedicine [190,191]. The P22 bacteriophage capsid naturally self-assembles from multiple copies of a capsid subunit with the aid of a scaffolding protein (SP). The C-terminus of the SP interacts noncovalently with the interior of the capsid, resulting in its incorporation, and this process guides the assembly of the capsid via the head-full mechanism. It has been shown that a completely modified SP protein in which the last few C-terminus residues are conserved still maintains the ability to lead the assembly of a native-like P22 capsid [42]. Also, SP-tagged proteins can be in vivo loaded into P22 [42]. Douglas and collaborators have used the P22 SP encapsulation method to load monomeric and oligomeric enzymes. The tetrameric β-glucosidase enzyme, CelB, and monomeric alcohol dehydrogenase D (AdhD) from *Pyrococcus furiosus* were individually loaded inside P22 VLP [115,145]. Additionally, this strategy has allowed for multiple enzymes, linked together with flexible spacers, to be collectively loaded inside P22 VLP. The co-encapsulated enzymes remained active and were able to perform a sequential enzymatic reaction in vitro (further details can be found in Section 5.2) [143].

Some PNPs do not have loading mechanisms (e.g., lumazine synthase and E2), or their native encapsulation mechanism is limited to a certain molecule (e.g., VLPs affinity to nucleic acids). In these cases, other genetic engineering strategies must be developed to facilitate the encapsulation of different cargo. A straightforward approach to encapsulate proteins inside a PNP was designed for lumazine synthase. The interior surface was engineered to provide a negatively charged environment inside the PNP by mutating four residues per monomer to glutamate. This resulted in the specific encapsulation of GFP carrying a positively charged tag (deca-arginine) at its C-terminus. This approach allows for the general encapsulation of other tagged proteins [192].

Other loading strategies are based on the specific interactions between two peptides. Giessen et al. used the well-known SpyTag/SpyCatcher system [193]—in which the peptide (SpyTag) forms an amide bond to its protein partner (SpyCatcher)—to package two enzymes involved in the biosynthesis of indigo dye inside MS2 [139]. Herein, SpyTag was expressed inside an MS2 capsid (SpyMS2), allowing the in vivo loading and covalent attachment of SpyCatcher-tagged enzymes [139]. Encapsulation of the two-enzyme indigo biosynthetic pathway improved the production of indigo dye in vivo (*E. coli*) and in vitro [139].

A similar loading approach was developed for CCMV VLPs. Usually, the loading of cargo inside CCMV is mainly performed by in vitro disassembly/reassembly with or without conjugation to DNA tags [118,120]. However, to control the encapsulation of enzymes inside CCMV, a noncovalent anchoring moiety was used to attach the target protein to the subunit before reassembly. A heterodimeric coiled-coil protein was employed as the anchor. The coiled-coil sequences were bound to the C-terminus of enhanced GFP (EGFP) (E-coil) and the N-terminus of CMMV capsid subunits (K-coil). The resulting EGFP–subunit complex was mixed with different concentrations of wild-type (wt) subunits to allow the reassembly of the capsid. This encapsulation method provided some control over the loading of EGFP per capsid by altering the ratios of wt and EGFP subunits and resulted in loads of up to 15 EGFP proteins per capsid [194].

#### 4.2.4. Interface Engineering

The self-assembly of PNPs relies on optimally balanced energetics that occur in part from the complex and dynamic interplay of amino acids at the interfaces between their subunits [195]. Understanding the underlying molecular mechanisms of self-assembly for supramolecular entities is complicated, particularly if their structures are highly symmetrical and homo-oligomeric, like most PNPs. To date, the self-assembly of PNPs has been investigated using various biophysical characterization techniques that rely on understanding the folding process of oligomeric proteins by chemical denaturation kinetics, as well as the calorimetric quantitation of thermal denaturation (e.g., differential scanning calorimetry, isothermal titration calorimetry, and pressure perturbation calorimetry). The chemical and thermal unfolding of protein oligomers can also be monitored by nuclear magnetic resonance and circular dichroism (CD) spectroscopy. These techniques in conjunction with structural and functional analysis, such as X-ray crystallography and mutation-related studies, can help elucidate the self-assembly and disassembly mechanisms of PNPs [196,197]. In-depth studies into the self-assembly of horse ferritin have revealed that subunits need to be completely folded to provide complementary interfaces for dimer formation. Based on these studies, a self-assembly mechanism for ferritin was proposed, in which dimers interact to form tetramers and hexamers. Subsequently, two hexamers can form one dodecamer, and two dodecamers self-assemble into the final 24-mer structure [196]. Studies on the pH disassembly of ferritin show that the 24-mer structure becomes unstable at pH 3.4. Different intermediate structures are formed during the dissociation process, starting with the formation of particles with two holes, followed by headphone-like structures, and finally resulting in mostly trimers and some monomers. Increasing the pH to neutral allowed the reassembly process of a nanoparticle with two hole defects [198].

As previously discussed in Section 4.2.3 in vitro disassembly processes have been exploited for cargo loading. The disassembly of certain PNPs can be initiated by pH [50,198], metals [199], reducing agents [130], ionic strength [119], or a combination thereof [117]. For example, encapsulin has been shown to disassemble in vitro under highly acidic or alkaline conditions, as well as in presence of high concentrations of denaturing agents (e.g., 7 M guanidine hydrochloride) [50]. To trigger their subsequent reassembly, optimal assembly conditions needed to be re-established (e.g., through dialysis). It has also been shown that the pH-driven disassembly/reassembly properties of PNPs can be altered by genetically modifying the interface between their subunits. For instance, ferritin disassembles at pH 2 and reassembles at pH 7. Such acidic pH can be a harsh condition for drug loading, especially when most bioactive compounds are unstable at extremely acidic pH. To allow a more amenable environment for cargo-loading, Chen et al. modified the 4-fold axis interface in ferritin by cleaving the last 23 amino acids at the C-terminus. This modified ferritin disassembled at a pH of 4.0 and was used to successfully encapsulate the bioactive compound curcumin inside ferritin [58].

The localized and controlled release of a drug enhances its therapeutic efficacy while reducing its harmful side effects. However, drug release from PNP cavities tends to be poorly controlled, working by simple diffusion and/or biodegradation. The ability to disassemble in specific physiological conditions is a crucial property required in the design of PNP-based drug delivery systems (DDSs). PNPs have been engineered to disassemble at acidic pH (5.0), which causes the release of their cargo upon cell internalization via endocytosis [151]. As an example, amino acids located at the N-terminus of E2 were deleted to cluster histidines at the interface between subunits. Repulsive interactions between histidines at acidic pH resulted in the destabilization of the E2 macrostructure [151]. E2 has also been modified to exhibit an inverse pH-sensitive self-assembly [200]. The synthetic GALA peptide, which consists of variable amino acid repeats of Glu-Ala-Leu-Ala, switches its conformation with changes in pH. GALA was used to replace a C-terminal α-helix known to be required for self-assembly of the E2 macrostructure. At pH 7.0, the GALA peptide is in the extended coil form, and thus, the modified E2 is disassembled. Lowering the pH to 4.0 switched GALA to its helix form, allowing the reassembly of the PNP. This process was shown to be to be reversible [200].

## 5. Application of Protein-Based Nanoparticles in Biomedicine and Biotechnology

In this section, we describe selected examples that show the progress made thus far in the development of PNPs as platforms for vaccine development, drug delivery, and biocatalysis. The reader is referred to other reviews for more information about the potential applications of PNPs in other fields, such as diagnostic imaging [201], biomineralization [202], and nanomaterial synthesis [203].

### 5.1. Biomedical Applications of Protein-Based Nanoparticles 

#### 5.1.1. Vaccine Development

Natural and synthetic PNPs have potential use in biomedicine as vaccines and as DDSs. While modifications on the outside of PNPs have been shown to be a viable strategy for vaccine development, the encapsulation of target molecules inside PNPs remains a more challenging option. For example, VLPs have been successfully developed and introduced as vaccines. In these VLPs, which lack genetic material, the outer surface is chemically or genetically modified to introduce a large number of anchoring points to which antigens can be connected to increase their immunogenicity [204]. In another example, HBc particles, which have the ability to serve as carriers of foreign B cell and cytotoxic T lymphocytes epitopes [205], have been used to genetically display antigens for malaria [206], tuberculosis [207], human papillomavirus (HPV) 16 cytotoxic T lymphocytes epitope E7 [208], and dengue virus type 2 [209]. Few VLP-based vaccines are commercially available worldwide, including vaccines against HPV (Gardasil^®^, Cervarix^©^, and Gardasil9^®^) and hepatitis B virus (Engerix^®^ and third-generation Sci-B-Vac™). Other VLP-based vaccine candidates are in different stages of preclinical and clinical trials [15,210].

**Figure 3:**
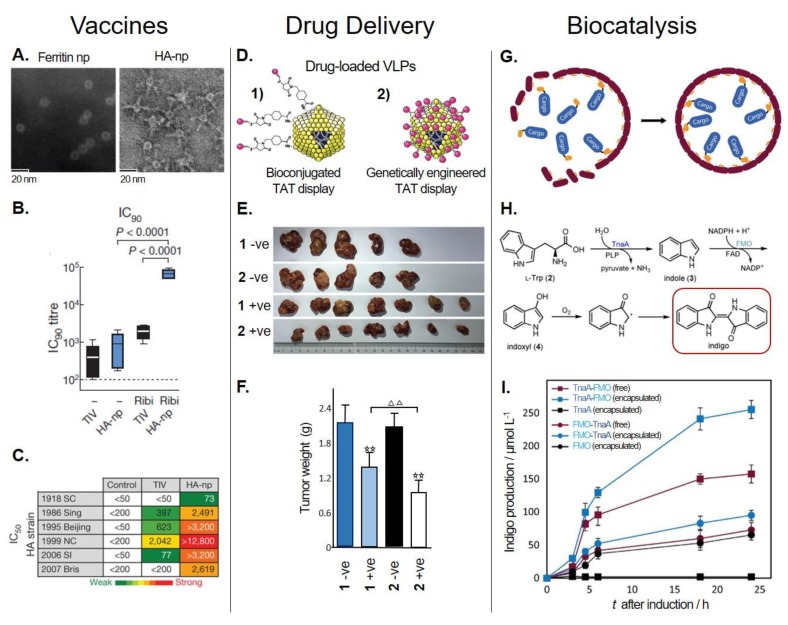
Examples of the bioapplications of PNPs. (**Left panel**) Vaccines: Development of a PNP-based influenza vaccine*.* (**A**) Transmission electron microscopy (TEM) image showing unmodified ferritin nanoparticles (np) (left) and modified ferritin np with visible hemagglutinin (HA) spikes (HA-np) (right). (**B**) Comparison of the immunogenicity (in mice) of trivalent inactivated influenza vaccine (TIV, control) or HA-np, without (−) or with adjuvant (Ribi)*.* Neutralization titers (IC_90_) were determined by measuring the concentration of antibody required to inhibit viral entry by 90%. (**C**) Table showing the neutralization (IC_50_ values) of immune sera induced by TIV or HA-np (with Ribi) against a range of H1N1 pseudotyped influenza viruses. Heat map is a colored gradient, from green (weak) to yellow to red (strong), reflecting the neutralization strength. Adapted with permission of [162]*.* (**Middle panel**) Drug delivery: A PNP-based microRNA delivery system for targeted cancer therapy. (**D**) MS2 VLPs loaded with an anticancer microRNA (miR122) and modified to display a cell-penetrating peptide (TAT) by either (**1**) bioconjugation (crosslinking); or (**2**) genetic engineering. (**E**) Tumors isolated from mouse models of hepatocellular carcinoma (HCC) after three weeks of treatment with either of the modified VLPs, which were loaded with miR122 (+ve) or a noncoding miRNA control (−ve). (**F**) Tumor weights after treatment with modified VLPs. Data compared with their negative controls (−ve) are represented by: ☆☆ *p* < 0.01; data from the genetically modified VLPs (2 +ve) treated group compared with the bioconjugated (crosslinked) VLPs treated group (1 +ve) are represented by: ΔΔ *p* < 0.01. Adapted with permission of [92]*.* (**Right panel**) Biocatalysis*:* In vivo loading of multiple enzymes inside PNPs for biocatalysis*.* (**G**) Genetic incorporation of the SpyCatcher/SpyTag system into the internal cavity of the MS2 VLP, which facilitates the loading of cargo tagged with either SpyCatcher or SpyTag during MS2 self-assembly in vivo. (**H**) Diagram of the sequential two-enzyme (i.e., pyridoxal phosphate (PLP)-dependent tryptophanase (TnaA) and flavin mononucleotide and nicotinamide adenine dinucleotide phosphate (NADPH)-dependent monooxygenase (FMO)) biosynthetic pathway for the production of indigo from l-tryptophan (l-Trp). (**I**) In vivo indigo production of *E. coli* strains expressing either of the encapsulated polycistronic operons (containing both enzymes): “TnaA + FMO” or “FMO + TnaA”, and their respective controls: free and single encapsulated enzymes. Adapted with permission of [139].

Nonviral PNPs can also be modified to display immunogenic epitopes that can be used potentially for vaccine development and immunotherapy [181,182]. One good example is *Helicobacter pylori* ferritin. Structural analysis of this protein revealed that the insertion of a heterologous protein—the influenza virus haemagglutinin (HA) subunit—near the N-terminus would allow HA to assume the physiologically relevant trimeric viral spike. The resulting fusion protein, ferritin–HA, was expressed in mammalian cells to achieve the glycosylation and post-translational modifications characteristic of viral proteins. Figure 3A shows transmission electron microscopy (TEM) images of ferritin–HA with spikes protruding from the spherical PNP. The protective immune response of ferritin–HA was compared with that obtained with trivalent inactivated influenza vaccine (TIV) in mice and ferrets. In the presence of the adjuvant Ribi, immune response indicators, such as hemagglutination inhibition (HAI), neutralization (IC_90_, Figure 3B) and ELISA titers, were significantly increased with ferritin–HA in both animal models. Additionally, the sera of immunized animals were used to assess the extent of neutralization against a panel of H1N1 pseudotyped viruses. Ferritin–HA elicited a major increment in influenza protection compared to the commercial vaccine (TIV, Figure 3C) [162]. Recently, this same platform was used to display HA stem-only (stem being the conserved region of HA glycoprotein) immunogens. Six iterative cycles of structure-based design were necessary to achieve H1HA stabilized stem immunogens that were later fused to ferritin. The HA stem-only ferritin vaccine exhibited antibody-mediated cross-immune protection against the H5H1 virus in mice and ferrets. This work represents an interesting platform for the potential development of universal influenza vaccines [163].

#### 5.1.2. Drug Delivery Systems

Conventional drug-based therapy lacks targeting and proper biodistribution, resulting in undesired side effects and a reduction in clinical efficacy [211]. In order to avoid these complications and provide an effective and localized treatment to patients, drugs must be targeted specifically, distributed, and controllably released at their primary site-of-action (e.g., tumors).

Drug delivery systems aim to minimize these limitations by altering a drug’s solubility, pharmacokinetics, and biodistribution [212]. Emerging nanotechnologies, in particular, NPs, show great promise as DDSs for cancer treatment [213]. Yet, very few synthetic NPs have advanced to become approved clinical therapies. This is due to their significant limitations, which include physicochemical heterogeneity, problematic functionalization, instability in physiological solutions, poor tumor penetration, and toxicity in biological systems [214]. The use of PNPs as nanocarriers is a promising alternative to synthetic NPs for the development of “smart” delivery systems. PNPs can be designed and produced to trigger the release of their cargo in response to changes in pH, chemical stimuli, redox potential, and temperature [215]. Most of the development of PNP delivery systems have focused on the treatment of cancer and the loading of cancer drugs inside PNPs in combination with targeting peptides. For example, in vitro studies showed no significant difference in the cytotoxic effect of RGD-targeted DOX-loaded HBc (RGD-HBc-NS5A/dox), DOX-loaded HBc without RGD (HBc-NS5A/dox), and free DOX. However, in vivo assays performed on mice xenografted with B16F10 tumors showed that RGD-HBc-NS5A/dox had significantly higher tumor growth inhibition (90.7%) compared to HBc-NS5A/dox (78.5%) and free DOX (72.1%). Furthermore, in vivo (mice) assessment of the potential systematic toxicity of RGD-HBc-NS5A/dox indicated that these nanocarriers were safe and displayed no observable hepatoxicity or nephrotoxicity [134].

Other therapeutic molecules that have been loaded inside PNPs include miRNAs. miRNAs are endogenous noncoding RNAs that can negatively regulate the gene expression of specific messenger RNAs (mRNAs) by inhibiting their translation or degradation. miRNA interference has been shown to have therapeutic properties in cancer and other diseases [216]. MS2 VLPs have been used as a vehicle for the targeted delivery of miRNA. For instance, MS2 was loaded with miR146a (MS2-miR146a), while the human immunodeficiency virus (HIV-1) cell-penetrating peptide (CPP) TAT was chemically crosslinked to its outer surface to facilitate uptake by different cell lines. The delivery system was able to induce miRNA expression both in vitro and in vivo [90]. The MS2-miR146a platform has shown other therapeutic effects by delaying the progression of systemic lupus erythematosus in mice [138] and repressing osteoclast differentiation [217].

This platform was improved further by Wang et al., who used phage surface display to genetically incorporate the HIV-1 TAT peptide and load another type of therapeutic miRNA (miR122) inside MS2 for the treatment of HCC [92]. The miR122-MS2 displaying TAT, by either bioconjugation (crosslinking) or genetic engineering, was evaluated for its therapeutic performance (Figure 3D). This delivery system was able to cross the cellular membrane and efficiently deliver miR122 to cancer cell lines (Hep3B, HepG2, and Huh7 HCC). Also, it was observed that the inhibition of invasion and induction of apoptotic rate in vitro, as well as tumor growth, weight, and proliferative capability in vivo (mice), showed significantly better results (*p* < 0.01 or 0.05) when using MS2-miR122 with the genetically incorporated TAT CPP compared to the chemically crosslinked variant (Figure 3E,F). This could be explained by the constant copy number of subunits forming the MS2 VLP, resulting in equal numbers of TAT peptides expected on the VLP’s surface. On the other hand, the TAT peptides bioconjugated on the native MS2 VLP relies on a more unstable crosslinking chemistry that may result in variable amounts of TAT on the VLPs. Therefore, a higher amount of evenly distributed TAT peptides could increase the efficiency of MS2 VLP uptake, causing a better inhibitory effect [92].

### 5.2. Biocatalysis

In nature, enzymatic pathways are compartmentalized into subcellular lipid-based structures or small protein-based compartments [115]. They contribute to confining enzymes and substrates in close proximity to allow faster conversion rates, protecting enzymes from degradation or inactivation, keeping toxic byproducts from damaging cell functions, and maintaining metabolic homeostasis [115]. Encapsulation of enzymes inside engineered PNPs is a novel approach towards fabricating catalytically active nanomaterials with unique properties [115]. Many PNPs have been used to encapsulate enzymes and perform catalytic reactions that in vivo would otherwise produce toxic or highly unstable chemicals. For example, the enzyme Aro10p catalyzes the decarboxylation of 4-hydroxyphenylpyruvate (4-HPP) to 4-hydroxyphenylacetaldehyde (4-HIPAA) [218]. 4-HIPAA can further react with dopamine via a cyclization reaction to form norcoclaurine, an intermediate for the production of benzylisoquinoline alkaloids [219,220]. However, 4-HIPAA production in yeast is challenging due the toxicity effects associated with aldehyde reactivity [221,222]. To address this limitation, Lau et al. encapsulated Aro10p inside encapsulin. The resultant encapsulin–Aro10p complex showed enzymatic activity with 4-HPP in vitro, resulting in spontaneous cyclization of 4-HPAA with dopamine to produce norcoclaurine [51].

Encapsulation can be applied also as a strategy to increase enzyme stability. Accordingly, manganese peroxidase (MnP)—an enzyme important in the bioremediation of water contaminated with aromatic compounds—was encapsulated inside vault PNPs. The enzyme was encapsulated via fusion to the mINT domain that interacts with the surface inside the vault. The vault-encapsulated MnP not only retained peroxidase activity, but they also displayed higher phenol biodegradation and higher stability towards thermal inactivation when compared to the free enzyme [187].

Some PNPs, such as VLPs, have been studied extensively to exploit their potential as catalytic nanoreactors. For example, Douglas and co-workers made use of synthetic biology tools to develop P22 as a platform for biocatalysis. They showed that P22 was able to encapsulate the tetrameric protein CelB—a β-glucosidase that hydrolyzes a wide variety of beta-linked disaccharides—from the hyperthermophile *P. furiosus*. The in vivo assembly and encapsulation resulted in P22 VLPs within high numbers of tetrameric CelB (~80 monomers per capsid). This high packaging density of CelB had no effect on the enzyme’s overall activity [115].

Recently, the P22 VLP system was developed further to achieve the efficient co-encapsulation of a multi-enzyme system capable of performing a coupled cascade of reactions [143]. The selected enzymes carry out sequential reactions in the sugar metabolism of the archaeon *P. furiosus*. The first enzyme is CelB, the second enzyme is the monomeric ATP-dependent galactokinase (GALK), and the third enzyme is the dimeric ADP-dependent glucokinase (GLUK). GALK and GLUK phosphorylate galactose to galactose-1-phosphate, and glucose to glucose-6-phosphate, respectively [223,224]. The design of the multi-enzyme system focused on the ability of CelB to break down lactose into galactose and glucose, which are then used as substrates for the other two enzymes. Additionally, ATP can be coupled to be a cofactor for both GALK and GLUK (once ADP is formed). For co-encapsulation of the enzymes, a gene construct was generated containing the enzymes linked together via flexible spacers. The fusion allows for co-encapsulation of enzymes in a defined ratio. Two protein fusions were evaluated and the effect of gene positioning inside the fusion was assessed, one containing two enzymes (CelB and GLUK) and the other with three enzymes (CelB, GLUK, and GALK). The encapsulated CelB-GLUK construct (CelB-GLUK-P22) showed no difference in enzyme activities compared to the single encapsulated enzymes (GLUK-P22 and CelB-P22, respectively). However, GLUK-CelB-P22 exhibited eight-fold CelB activity reduction compared to CelB-P22. Also, the production yield of GLUK-CelB-P22 decreased two-fold in comparison to CelB or GLUK-P22. These results indicate that the arrangement of enzymes within the fusion can have important effects on both VLP assembly and enzyme activity. The two and three co-encapsulated enzymes were able to perform the cascade reaction, showing similar kinetic parameters to the results achieved by a mixture of equal ratios of single encapsulated enzymes [143].

Enzyme catalytic rate declines have been reported also after encapsulation. Rate declines have been attributed to macromolecular crowding, protein conformational change restrictions during the enzymatic reaction, diffusion of product from the enzyme–substrate complex, and differences in activity coefficients between a native enzyme and an enzyme–substrate complex [42,225]. Also, the colocalization of enzymes does not always achieve enhanced yield activities. Therefore, understanding how the balance of each enzyme’s kinetic parameters and the interenzyme distance affect the flux of intermediates in an enzymatic cascade plays an important role in the design of more complex synthetic pathways [143].

The above examples were performed in vitro at small scales, and they required purification of all the components involved in the reaction. However, in vivo encapsulation and production seems to be better suited for a low-cost industrial manufacture of chemicals at large scale. For example, Giessen et al. encapsulated the two-enzyme biosynthetic pathway for the commercially relevant indigo dye inside MS2 (Figure 3G) [139]. The enzymes were packaged inside MS2 using the previously described SpyTag/SpyCatcher system (Section 4.2.3). During the biosynthetic pathway of indigo dye (Figure 3H), the pyridoxal phosphate (PLP)-dependent tryptophanase (TnaA) converts l-tryptophan to indole, which is oxidized to indoxyl by the flavin mononucleotide and nicotinamide adenine dinucleotide phosphate (NADPH)-dependent monooxygenase (FMO). Deep blue indigo is formed by the dimerization of indoxyl in the presence of molecular oxygen [226,227]. Two different polycistronic constructions were assessed (TnaA-FMO and FMO-TnaA) and the production of indigo dye was evaluated in vitro and in vivo (*E. coli*). In vivo production was compared between strains expressing the SpyMS2 (encapsulated) or the wt MS2 (wtMS2, free) along the pathway. The order of the genes in the constructions was found to be very important. As shown in Figure 3I, when the pathway was encapsulated, the TnaA-FMO strain outperformed the FMO-TnaA strain, achieving 100% higher production of indigo dye. Also, the production of indigo dye with encapsulated enzymes showed a 60% increase in indigo dye production despite their slightly lower amount of soluble TnaA/FMO compared to the wtMS2 strain (Figure 3I). Furthermore, the encapsulated enzymes displayed higher stability and maintained their initial activity at 25 °C for seven days, whereas free enzymes lost more than 90% of their initial activities under these conditions. The improvement in production of indigo dye could be attributed to the increased local concentration of intermediates and enhancement of enzyme stability upon encapsulation [139].

## 6. Conclusions and Future Perspectives

Protein-based nanoparticles have the capacity to be re-engineered, with countless reports detailing the attachment of a diverse range of functional small molecules and macromolecules to their inner and outer surfaces. Various genetic modification and/or bioconjugation techniques have been established to develop PNP-based platforms for applications in drug delivery, nanomaterial synthesis, vaccines, and biocatalysis [12]. However, predicting the effects these modifications have on a PNPs’ structural stability and its ability to self-assemble is an ongoing challenge.

The interfaces between subunits are the most poorly understood PNP surface, but arguably the most important and promising one, as PNP self-assembly, disassembly, and reassembly, and also their surface pores, are dependent upon it. Thus, unraveling the underlying mechanisms of PNP self-assembly and the processes driving pore opening and closing is essential for the controlled loading/release and the transfer of cargo molecules in and out of the PNP’s cavity. This knowledge will expedite the translation of PNPs into drug delivery applications, in which spatial and temporal control of the release of a drug enhances its therapeutic efficacy and also minimizes any harmful off-target side effects. Additionally, this information can help create more efficient synthetic biocatalytic nanoreactors by enabling better regulation of substrate and/or product flux through PNP surface pores.

Finally, new in silico tools that allow the accurate prediction of protein structure and assembly, in combination with high-throughput characterization techniques, will pave the way towards the production of functional PNPs that are tailored to their ultimate application.

## Figures and Tables

**Table 1:** Function, structure, and production of the most commonly used protein-based nanoparticles (PNPs).

PNP		Native Organism	Biological Function	Geometry	Number of Subunits	Size (Diameter)	Heterologous Production	Ref.
Virus-like particles (VLPs)	CCMV ^1^	Cowpea chlorotic mottle virus capsid protein	Plant virus	Icosahedral	182	28 nm	Plants; yeast; *Escherichia coli; Pseudomonas fluorescens*	[11,28,29,30]
	CPMV ^2^	Cowpea mosaic virus capsid protein	Plant virus	Pseudo icosahedral	120 Large (L) and 120 Small (S)	28 nm	Insect cells; plants	[31,32]
	HBc ^3^	Hepatitis B virus capsid protein	Human virus	Icosahedral	180 or 240	30 nm or 34 nm	Mammalian cells; insect cells; plants; yeast; *E. coli*; cell-free	[33,34,35,36,37,38]
	MS2	Enterobacteriaceae	Bacteriophage	Icosahedral	180	26 nm	Yeast; *E. coli*; cell-free	[39,40,41]
	P22	*Salmonella typhimurium*	Bacteriophage	Icosahedral	420	60 nm	*E. coli*	[42]
	Qβ	*E. coli*	Bacteriophage	Icosahedral	180	28 nm	Yeast; *E. coli*; cell-free	[43,44,45]
Non-viral PNPs	Dps ^4^ (mini-ferritin)	Archaea; Bacteria (e.g., *Listeria innocua*)	Involved in oxidative and starvation responses	Tetrahedral	12	9 nm	*E. coli*	[46,47]
	E2	*Bacillus stearothermophilus*	Core of the pyruvate dehydrogenase multienzyme complex	Dodecahedral	60	24 nm	*E. coli*	[48]
	Encapsulin	Archaea; Bacteria	Involved in oxidative stress response	Icosahedral	60 or 180	20–40 nm	Mammalian cells; yeast; *E. coli*	[49,50,51,52]
	Ferritin (maxi-ferritin)	Archaea; Bacteria; Eukarya	Iron storage	Octahedral	24	12 nm	Mammalian cells; insect cells; yeast; *E. coli*	[53,54,55,56,57,58]
	Hsp ^5^	Archaea; Bacteria; Eukarya (e.g., *Methanococcus jannaschii*)	Chaperone	Octahedral	24	12 nm	*E. coli*	[59,60]
	Lumazine synthase	Archaea; Bacteria; Eukarya (e.g., *Aquifex aeolicus*)	Mediates the biosynthesis of riboflavin	Icosahedral	60	15.4 nm	*E. coli*	[61,62]
	Vault protein	Eukarya	Involved in signaling and immune responses	39-fold dihedral	78 Major vault protein	Diameter: 40 nm Length: 67 nm	Insect cells; cell-free	[63,64,65,66]

^1^ Cowpea chlorotic mottle virus, ^2^ Cowpea mosaic virus, ^3^ Hepatitis virus, ^4^ DNA-binding proteins from starved cells, ^5^ Heat shock proteins.

**Table 2:** Methods used to bioengineer the most commonly used PNPs for a range of bioapplications.

	PNP	In Vitro Loading Mechanism	Cov Biocon ^1^	Point Mut ^2^	UAA ^3^	Pep Disp ^4^	Prot Disp ^5^	Modul Assem ^6^	Encapsulated Cargo			Applications	Ref.
									Diffusion ^7^	In Vitro	In Vivo		
**VLPs**	CCMV	pH; Ionic strength	•	•		•	•			Metals; small-molecule drugs; nucleic acids; organic polymers		Drug delivery; vaccines; bioimaging; prodrug activation; biocatalysis	[30,117,118,119,120,121,122,123,124]
	CPMV		•	•		•	•		Metals; fluorescent probes; biotin; organic polymers			Drug delivery; vaccines; bioimaging	[31,91,125,126,127,128,129]
	HBc	Denaturants	•	•	•	•	•	•		Metals; small-molecule drugs; fluorescent probes; nucleic acids		Drug delivery; vaccines; bioimaging	[37,130,131,132,133,134,135]
	MS2	pH; Denaturants	•	•	•	•	•		Fluorescent probes; photosensitizers	Metals; small-molecule drugs; nucleic acids	Proteins	Drug delivery; vaccines; bioimaging; biocatalysis	[114,136,137,138,139,140]
	P22	pH	•	•		•		•	Metals; fluorescent probes; biotin; organometallic polymers	Proteins	Proteins; peptides; epitopes; nucleic acids	Drug delivery; vaccines; nanomaterial synthesis; biocatalysis; solubility enhancement	[107,141,142,143,144,145,146]
	Qβ	pH; Denaturants	•	•	•	•	•		Fluorescent probes; cationic polymers	Metals; small-molecule drugs; fluorescent probes; nucleic acids	Proteins	Drug delivery; vaccines; bioimaging; nanomaterial synthesis	[24,97,132,137,147,148]
	Dps		•			•		•	Metals			Drug delivery; nanomaterial synthesis	[104,149,150]
	E2	Denaturants	•	•		•	•	•	Small-molecule drugs; fluorescent probes	Nucleic acids		Drug delivery; vaccines; biocatalysis; antibody purification	[48,82,151,152,153,154,155,156]
	Encapsulin	pH; Denaturants	•	•		•	•		Metals	Proteins	Proteins	Drug delivery; bioimaging; immunotherapy; antimicrobials; biocatalysis	[9,50,51,52,157,158,159]
	Ferritin	pH	•	•	•	•		•	Metals; small-molecule drugs	Bioactive compounds; metals; small-molecule drugs	Metals	Solubility enhancement; drug delivery; vaccines; bioimaging; immunotherapy; nanomaterial synthesis	[27,102,160,161,162,163,164,165,166,167,168,169]
	Hsp	Temperature	•	•		•	•		Metals	Metals; small-molecule drugs; dyes; fluorescent probes		Drug delivery; nanomaterial synthesis; biocatalysis	[59,87,170,171,172]
	LS	Ionic strength	•	•		•	•	•		Proteins		Drug delivery; vaccines; bioimaging; biocatalysis	[62,98,173,174,175,176,177]
	Vault	“Breathing mechanism”	•			•				Metals; proteins; epitopes; antigens	Proteins	Solubility enhancement; drug delivery; vaccines; bioimaging; immunotherapy; bioremediation	[178,179,180,181,182,183,184,185,186,187]

^1^ Covalent bioconjugation; ^2^ Point Mutation; ^3^ Unnatural amino acid incorporation; ^4^ Peptide display; ^5^ Whole protein display; ^6^ Modular assembly; ^7^ Pore-mediated diffusion.
